# Lack of macrophage migration inhibitory factor in mice does not affect hallmarks of the inflammatory/immune response during the first week after stroke

**DOI:** 10.1186/1742-2094-8-75

**Published:** 2011-06-29

**Authors:** Ana R Inácio, Richard Bucala, Tomas Deierborg

**Affiliations:** 1 Laboratory for Experimental Brain Research, Department of Clinical Sciences, Lund University, BMC A13, 22184 Lund, Sweden; 2 Department of Medicine and Pathology, Yale University, New Haven, Connecticut, USA

**Keywords:** cluster of differentiation 74 (CD74), cytokines, glial fibrillary acidic protein (GFAP), galectin-3 (Gal-3)/Mac-2, macrophage migration inhibitory factor (MIF), transient middle cerebral artery occlusion (tMCAo)

## Abstract

**Background:**

Macrophage migration inhibitory factor (MIF) has been proposed to play a detrimental role in stroke. We recently showed that MIF promotes neuronal death and aggravates neurological deficits during the first week after experimental stroke, in mice. Since MIF regulates tissue inflammation, we studied the putative role of MIF in post-stroke inflammation.

**Methods:**

We subjected C57BL/6 mice, *Mif*^-/- ^(MIF-KO) or *Mif*^+/+ ^(WT), to a transient occlusion of the right middle cerebral artery (tMCAo) or sham-surgery. We studied MIF expression, GFAP expression and the number of CD74-positive cells in the ischemic brain hemisphere 7 days after tMCAo using primarily immunohistochemistry. We determined IFN-γ, IL-2, IL-4, IL-5, IL-10, IL-12, KC/CXCL-1 and TNF-α protein levels in the brain (48 h after surgery) and serum (48 h and 7 days after surgery) by a multiplex immunoassay.

**Results:**

We observed that MIF accumulates in neurons and astrocytes of the peri-infarct region, as well as in microglia/macrophages of the infarct core up to 7 days after stroke. Among the inflammatory mediators analyzed, we found a significant increase in cerebral IL-12 and KC levels after tMCAo, in comparison to sham-surgery. Importantly, the deletion of *Mif *did not significantly affect the levels of the cytokines evaluated, in the brain or serum. Moreover, the spleen weight 48 h and 7 days subsequent to tMCAo was similar in WT and MIF-KO mice. Finally, the extent of GFAP immunoreactivity and the number of MIF receptor (CD74)-positive cells within the ischemic brain hemisphere did not differ significantly between WT and MIF-KO mice subjected to tMCAo.

**Conclusions:**

We conclude that MIF does not affect major components of the inflammatory/immune response during the first week after experimental stroke. Based on present and previous evidence, we propose that the deleterious MIF-mediated effects in stroke depend primarily on an intraneuronal and/or interneuronal action.

## Background

Focal cerebral ischemia, experimentally induced in rodents and in the clinical setting, causes an early and sustained activation of inflammatory and immune cascades, both locally in the brain and outside the brain [1]. These cascades affect cerebral cell death and also are believed to integrate mechanisms of later functional recovery [2]. Dynamic alterations in the production/release of several pro and anti-inflammatory cytokines, including chemokines, in the brain and periphery are part of the inflammatory/immune response following stroke [3]. Interleukin (IL)1-β expression increases in the rodent brain subsequent to middle cerebral artery occlusion (MCAo), [4], both promoting cell death [5,6] and repressing recovery of neurological function [7]. Tumor necrosis factor (TNF)-α may exert both neurotoxic and neuroprotective effects, depending on its exact spatial-temporal expression profile [8]. In stroke patients, increased cerebrospinal fluid and/or circulating IL-1β, IL-6, IL-10, and CXCL-1 (CINC-1/IL-8), monocyte chemoattractant protein-1, and TNF-α concentrations have been reported [3], and IL-6 in particular identified as a predictor of stroke outcome [9]. Regulatory cytokine pathways and signaling may mediate or co-exist with glial activation/gliosis and the recruitment of peripheral immune cells to the locus of injury. The formation of an astroglial scar, typically during the first week after stroke, is generally believed to beneficially constrain damage expansion. Moreover, it may be necessary for or repress neuroregeneration and repair [10,11]. Similarly, detrimental and beneficial actions of activated microglia/invading macrophages [12-14] and other immune cells have been reported [15]. The inflammatory/immune response following stroke also is thought to comprise an induced peripheral immunodepression [16], given for instance by a shift from T helper cell (Th)1 to Th2 cytokine production [17] and splenic atrophy [18].

Macrophage Migration Inhibitory Factor (MIF) is a multifunctional, ubiquitous protein. Among its many identified modes of action, MIF is an upstream regulator of inflammatory-immune processes [19]. In particular, MIF has a pro-inflammatory action in local and systemic inflammatory and immune responses outside the brain, which can be highly detrimental, for example, in atherosclerosis, rheumatoid arthritis [20,21] and sepsis [22]. Here, multiple mechanisms are involved, among which MIF promotes the expression of pro-inflammatory cytokines [19], leukocyte adhesion and infiltration [21] and immune cell proliferation [23,24]. The role of MIF in the brain is far less known, but MIF may critically affect the inflammatory reaction under pathological conditions [25-27].

MIF is rapidly and persistently up-regulated around the infarct core after focal cerebral ischemia in rodents [28-30]. Moreover, MIF was shown to increase in the plasma of stroke patients within the first 3 days and positively correlated to the severity of neurological deficits [28]. We previously found that *Mif*^-/- ^(MIF-KO) mice have a smaller infarct volume than the respective wild-type (WT) littermates and perform significantly better when tested for sensory-motor deficits at 48 h and 7 days after transient MCAo (tMCAo), [29]. A similar outcome was found after spinal cord injury [31]. We further observed that environmental enrichment 2 to 5 days subsequent to permanent MCAo in rats down-regulates MIF in the peri-infarct region and, more distally, in the cingulate cortex, when compared to standard housing conditions [30]. Collectively, these results indicate that MIF promotes cell death and represses the recovery of neurological function in experimental central nervous system injury.

The up-regulation of MIF in the peri-infarct region occurs in neurons at least up to 72 hours after tMCAo [29]. Neuronal MIF expression per se promotes neuronal death and/or renders neuronal networking more susceptible to excitotoxic [31] and ischemia-like [29] conditions-induced death, and it may further affect plasticity events [30,32]. Whereas MIF acts as an upstream regulator of inflammatory/immune reactions after stroke remains to be determined.

Here, we quantified the expression of Th1/Th2 cytokines in the brain and serum, and the spleen weight of WT and MIF-KO mice during the first week after tMCAo. Additionally, we studied the effect of the genetic deletion of MIF in mice on reactive astrogliosis, and number of MIF receptor (CD74)-positive inflammatory cells within the injured brain hemisphere.

## Material and Methods

### Animal housing conditions and ethical considerations

Mice were housed in climate controlled rooms under a 12 h light-12 h dark cycle, with ad libitum access to water and food. Animal experiments were conducted according to protocols approved by the Malmö/Lund Ethical Committee for Animal Research.

### Strains and genotyping

We used 9 to 14 weeks old C57BL/6 male mice (purchased from Taconic, Denmark) only when characterizing the expression of MIF in the brain after transient middle cerebral artery occlusion (tMCAo).

To determine if MIF affects the inflammatory/immune response upon tMCAo, we used *Mif*^+/+ ^(WT) and *Mif*^-/- ^(MIF-KO) male mice, littermates, on a pure C57BL/6 background [33], 8 to 36 weeks old. These experiments were performed in a blinded and randomized fashion.

### Transient middle cerebral artery occlusion (tMCAo)

We induced focal cerebral ischemia in mice by occluding the right middle cerebral artery (MCA) for 45 min as described in [29]. In summary, mice were initially anesthetized by inhalation of 2.5% isoflurane (IsobaVet, Schering-Plough Animal Health, UK) in O_2_:N_2_O (30:70). Thereafter, mice were placed on a heating pad and anesthesia was subsequently reduced to and maintained at 1.5-1.8% (using an open mask). We used a rectal temperature probe to monitor body temperature, kept at approximately 37°C. An optical fiber probe (Probe 318-I, Perimed, Sweden) was firmly attached to the skull and connected to a laser Doppler flow meter (Periflux System 5000, Perimed), allowing us to monitor changes in local/regional cerebral blood flow (rCBF). We then inserted a filament composed of a 6-0 polydioxanone suture (PSD II, Ethicon, Germany) and a silicone tip with a diameter of 225 to 275 μm (depending on the age of the animal) into the lumen of the external carotid artery via a small wall incision. We advanced the filament up to the origin of the MCA, given by a sudden drop in rCBF. The filament was retracted 45 min after and reperfusion observed. Bupivacaine (0.150 μL, 0.05%, Marcain™, AstraZeneca, Sweden) was injected around the wound to reduce pain. In order to avoid post-surgical hypothermia, animals were placed in an incubator at 35°C for the first 2 hours after occlusion and then transferred to an incubator at 33°C (overnight). At 30 min and 24 h post-surgery, we administered 0.5 mL of 5% glucose in saline subcutaneously. In animals allowed to recover for 7 days, we further administered 0.5 mL of 5% glucose subcutaneously every 12^th ^hour up to day 4 post-surgery (which is when weight loss ceases). Body weight was controlled daily up to the experimental end-point. Additionally, we assessed temperature and gross sensory-motor deficits at 1 h, 2 h and 24 h after occlusion. In sham-surgeries, the filament was advanced up to the internal carotid artery, and withdrawn before reaching the MCA.

Mice that did not verify an immediate reduction in rCBF upon MCAo and reperfusion (approximately 8%) were immediately sacrificed and excluded from the study; intracerebral hemorrhage was confirmed in all the cases by analysis of 1 mm thick brain slices.

### 2,3,5-Triphenyltetrazolium chloride (TTC) staining

**Figure 1:**
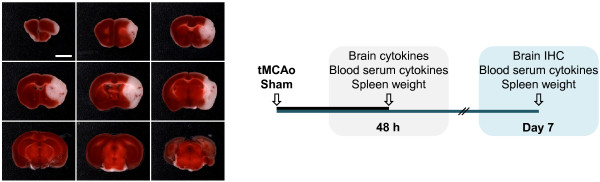
**Experimental settings**. **(A) **Composite photograph of a TTC stained brain 24 h after tMCAo. Representation of the cerebral damage obtained in average after a 45 min occlusion of the right middle cerebral artery, in C57BL/6 mice (white). Scale bar: 3 mm. **(B) **Experimental outline. IHC, immunohistochemistry.

To verify a reproducible damage after a 45 min tMCAo, WT mice (n = 8) were sacrificed at 24 h post-occlusion and brains were sliced into 1 mm thick sections, using a mouse brain matrix on ice. Sections were rinsed in ice-cold 0.9% NaCl for 10 min, and thereafter immersed in a aqueous solution composed of 0.01% TTC and 0.9% NaCl for 15 min at 37°C. Slices were fixed in 4% formaldehyde and pictures were acquired using a MicroPublisher 3.3 RTV CCD camera (QImaging, Canada), under standardized conditions. We encircled the contralateral hemisphere area, the ipsilateral hemisphere area and the affected area (lack of TTC staining; designated as infarct area for simplification) of each slice using ImageJ software (National Institutes of Health, USA). The infarct area (IA) was corrected for shrinkage, IAcorrected = Contra/Ipsi x IA, and the infarct volume was calculated by volumetric integration. We obtained infarct volumes of approximately 53.8 ± 24.5 mm^3 ^(mean ± SD), (Figure 1 A).

### Sample collection I (48 h)

We anesthetized WT and MIF-KO mice at 48 h after sham-surgery or tMCAo, by inhalation of 2.5% isoflurane. Blood (200-400 μL) was collected by heart puncture having a minimal flow resistance to avoid hemolysis. Thereafter, mice were perfused transcardially with 0.9% NaCl for 2 min and decapitated. Upon removal of the meninges, brains were quickly obtained and frozen by immersion in isopentane at -40°C, further cooled down to -70°C and stored at -80°C. Spleens were collected and weighed. Blood was allowed to coagulate for approximately 30 min. After standard centrifugation, blood serum was obtained and stored at -80°C. We dissected both the infarct core (IC) and the remaining tissue of the ipsilateral hemisphere, designated as non-infarct region (NIR) for simplification, from coronal brain sections, at -15°C. At this temperature and in view of a critical difference in tissue organization, IC and NIR can be easily detached. Specifically, we obtained consecutive 2 mm, 1 mm and 2 mm thick brain sections, starting at 2 mm from the olfactory bulb. In sham-operated mice, we dissected equivalent brain regions, also designated as IC and NIR. A schematic representation of the dissections is included in the Results section.

### Sample collection II (7 days)

At day 7 after tMCAo, C57BL/6 mice (Taconic), WT and MIF-KO mice were anesthetized and perfused transcardially with 0.9% NaCl for 2 min followed by 4% formaldehyde. For the WT and MIF-KO mice, we collected blood prior to saline/fixative perfusion; spleens were obtained and weighed. All the brains were kept in fixative overnight, transferred to 30% sucrose (in PBS), and subsequently cut into 30 μm-thick coronal sections (free-floating).

The experimental outline is presented in Figure 1 B.

### Protein Extraction and cytokines/chemokines immunoassay

Whole cellular protein extractions from the dissected IC and NIR were performed by mechanical homogenization and incubation in lysis buffer: 20 mM Tris (pH 7.5), 150 mM NaCl, 1 mM EDTA, 1 mM EGTA, 1% Triton-X100, 2.5 mM sodium pyrophosphate, 1 mM β-glycerolphosphate, 1 mM orthovanadate, 1 mM PMSF, supplemented with a protease inhibitor cocktail (P8340, Sigma-Aldrich, Germany). Following 30 min incubation, samples were centrifuged at 18000 xg, for 15 min. Total protein concentration was determined by the Bradford assay, using bovine albumin (Sigma-Aldrich) as standard. Interferon (IFN)-γ, interleukin (IL)-10, IL-12, IL-1β, IL-2, IL-4, IL-5, tumor necrosis factor (TNF)-α and keratinocyte-derived cytokine (KC)/CXCL1 protein levels were determined by a sandwich immunoassay essentially according to the manufacturer's recommendations (ultra-sensitive protocol, Meso Scale Discovery, USA) and using a SECTOR Imager 6000 (Meso Scale Discovery). A few modifications were introduced: for assays including brain samples, standards were dissolved in lysis buffer; brain extracts and serum were incubated with the capture antibody for 4 h. The results correspond to the protein concentration (pg/mL) when using 150 μg of protein per brain sample (in 50 μL per well); serum was assayed undiluted (30 μL per well).

### General immunohistochemistry

Formaldehyde-fixed free-floating brain slices were rinsed three times in phosphate-buffered saline (PBS) and thereafter kept in a 5% blocking solution (5% normal serum, Jackson ImmunoResearch, UK, and 0.25% Triton X-100 in PBS) for 1 h at room temperature. Sections were then incubated with primary antibody/antibodies diluted in 2% blocking solution overnight at 4°C (Table 1). Next, sections were rinsed three times (in PBS) and incubated with secondary antibody/antibodies conjugated with the fluorescent dyes Cy3, Cy5 (Jackson ImmunoResearch) and Alexa488 (Molecular Probes, Invitrogen, UK), except when using a GFAP-Cy3 direct conjugate. When detecting the primary antibody by the biotin-avidin-horseradish peroxidase system (VECTASTAIN Elite ABC kit, Vector Laboratories, UK), brain sections were quenched in 3% H_2_O_2_/10% methanol for 15 min and rinsed prior to blocking. We used all the secondary antibodies at a dilution of 1:400. Fluorescence was detected by laser-scanning microscopy (LSM 510, Zeiss, Germany), except when indicated, using equal settings among experimental conditions. We acquired bright-field micrographs with an Olympus BX60 microscope (Olympus, Sweden), except when indicated, using standard conditions among sections.

**Table 1:** Primary antibodies

Primary antibody	Supplier	Catalogue	Dilution
Rabbit anti-MIF	Torrey Pines Biolabs	TP234	1:300-1:5000

Mouse anti-Tuj1	Chemicon (Millipore)	MAB1637	1:500-1:800

Mouse anti-Parvalbumin	Sigma-Aldrich	P3088	1:4000-1:6000

Mouse anti-GST-p	BD Transduction Laboratories	610718	1:500

Rat anti-Mac2, biotinylated	Acris Antibodies	CL049B	1:500

Mouse anti-GFAP, Cy3	Sigma-Aldrich	C9205	1:5000

Mouse anti-GFAP	Sigma-Aldrich	3893	1:2500

Rat anti-CD31	BD Pharmingen	550274	1:200

Rat anti-CD74	BD Pharmingen	555317	1:200

### GFAP and CD74 immunohistochemistry

We detected GFAP and CD74 in brain sections of WT and MIF-KO mice sacrificed 7 days after tMCAo by immunohistochemistry. We used a monoclonal anti-GFAP antibody (1:2500, Sigma-Aldrich) and suitable Cy3-conjugated secondary antibody; the anti-CD74 antibody (1:200, BD Pharmingen, Sweden) was visualized by the biotin-avidin-horseradish peroxidase system. We used three brain sections per animal, corresponding approximately to the following levels: 1.18, 0.50 and -1.82 mm, in relation to bregma. Accordingly, fluorescence and bright field micrographs of the ipsilateral brain hemisphere represented in each section were acquired (in grayscale) under a 10X objective, using a Nikon Eclipse 80i microscope and the NIS-Elements software (Nikon, Solna, Sweden). Pictures were acquired using the same settings, respectively. Final composite images were subsequently generated by NIS-Elements software.

### Analysis of the cerebral GFAP immunoreactivity

When analyzing cerebral GFAP immunoreactivity (GFAP-ir), we considered the following parameters: 1) GFAP^+ ^area within each composite image, excluding the hippocampus (encircled area), 2) signal intensity within the encircled area, 3) signal intensity within the ipsilateral hippocampus, and 4) cortical and subcortical widths of the GFAP-ir. In particular, we measured four cortical widths. For that purpose, we considered four virtual lines (from the top to the bottom of the cortex), dividing the cortex in approximately three equal parts; these lines intercepted the cortical infarct core/peri-infarct core border zone (virtual line) in an angle similar to that of the natural orientation of the cortical layers. We determined the subcortical width at the ventral-dorsal level 2.8 mm (close to the ventricle wall) and 1.0 (nucleus accumbens). Two persons blinded to the animal groups evaluated all the parameters independently.

### CD74 counts

We estimated the number of CD74^+ ^cells within each section by 1) encircling the CD74^+ ^area within each composite image (roughly, the infarct core), 2) setting a signal intensity threshold for each composite image, and 3) an automated counting of CD74^+ ^particles, using NIS-Elements software. We present the results as the total number of particles detected per brain. The light conditions at acquisition across images (background) were evaluated prior to setting the threshold; four slightly different thresholds were used according to the specific image background. Importantly, the background did not differ significantly between WT and MIF-KO mice neither did the selected threshold level. Given that MIF-KO mice have smaller infarct volumes than WT littermates [29], we further analyzed the *particles area *and the fraction *particles area/encircled area *per section. We also analyzed CD74-ir in terms of signal intensity within the encircled area, signal intensity of the selection upon setting the threshold, and mean intensity per particle.

### Statistics

Data are presented as means ± standard deviation (SD), unless otherwise specified. For all the statistical tests, results were considered significant when p < 0.05. The concentration of each of the inflammatory mediators included in this study, as well as the IC, NIR and serum were analyzed independently. Differences among groups (WT and MIF-KO mice, subjected to either sham-surgery or tMCAo), were assessed by 2-way analysis of variance and post-hoc by the Tamhane's test. With respect to the GFAP and CD74 evaluations, each of the parameters included in the experimental design was tested separately. To compare respective differences between the two experimental groups (WT and MIF-KO mice subjected to tMCAo) we used the Student's t-test. For each parameter, we further evaluated putative brain level-specific differences (between WT and MIF-KO mice) by the Student's t-test.

## Results

### Cerebral MIF expression in C57BL/6 mice 7 days after tMCAo

We previously characterized the spatial-temporal expression of MIF (protein) in the brains of C57BL/6 mice up to 72 h after 45 min tMCAo. In the present study, we further investigated the expression of MIF (protein) in the brains of C57BL/6 mice 7 days after 45 min tMCAo, by immunohistochemistry (n = 2-13).

**Figure 2:**
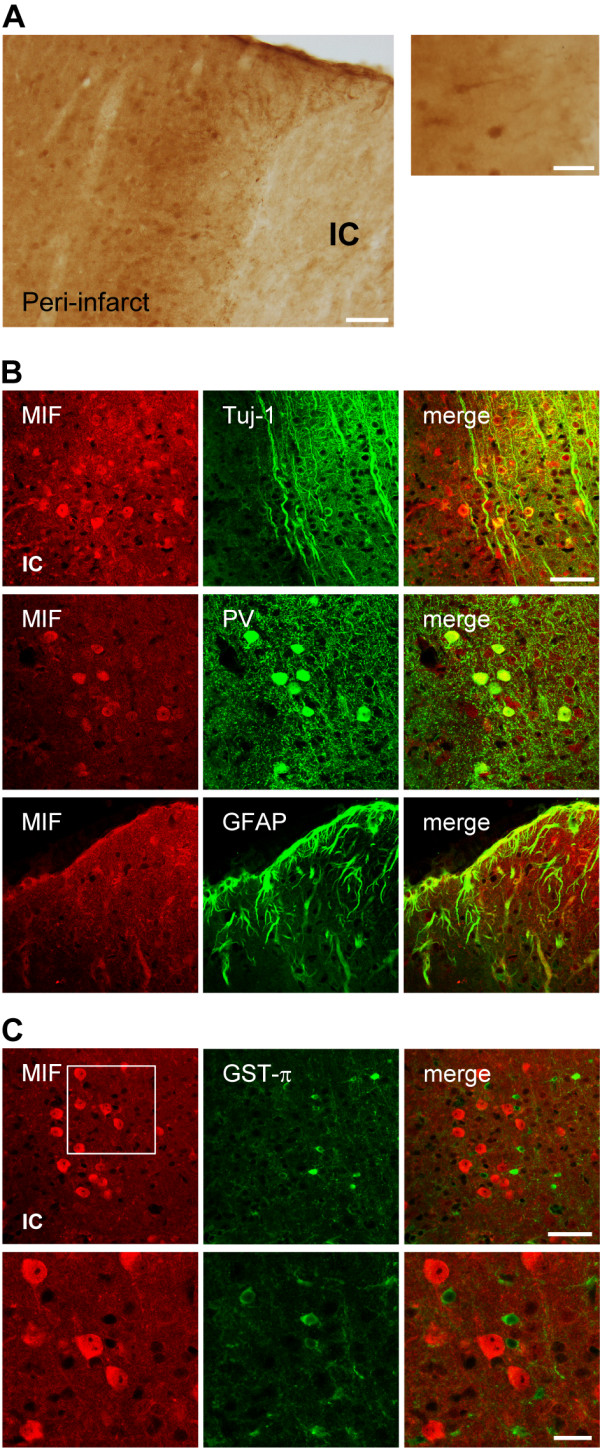
**MIF accumulates in neurons and astrocytes of the peri-infarct region during the first 7 days after tMCAo, in C57BL/6 mice**. **(A) **Bright field micrographs. Representative image of MIF immunoreactivity (MIF-ir) in the peri-infarct motor/somatosensory cortex at 7 days after tMCAo. Scale bar: 100 μm (right). A higher magnification image denotes the expression in neurons and glia-like cells. Note the thick glia-like processes positive for MIF around the infarct core. Scale bar: 50 μm (left). **(B) **Confocal micrographs. Co-localization of MIF (Cy3, red) with the neuronal markers Tuj-1 and PV (both Cy5, green). Co-localization of MIF (Cy3, red) and the marker GFAP (Cy5, green) confirms the presence in reactive astrocytes. Scale bar: 50 μm. Infarct core-IC. **(C) **Confocal micrographs. Lack of co-localization of MIF (Cy3, red) and GST-π (Cy5, green) in the peri-infarct somatosensory-motor cortex at 7 days after occlusion. Scale bars: 50 μm and 20 μm (lower and higher magnification, respectively).

Within the first 72 h post-occlusion, MIF immunoreactivity (MIF-ir) increased in the peri-infarct region, particularly in the peri-infarct cortex. Somewhat surprisingly, this increase occurred primarily in neurons, including parvalbumin-containing interneurons [29]. Between 72 h and 7 days after tMCAo, the cellular distribution of MIF in the peri-infarct region changed markedly. Figure 2 A exemplifies the observed expression of MIF in the motor/somatosensory cortex at day 7 post-occlusion. At this time-point, MIF-ir remained relatively elevated in Tuj-1^+ ^cells (neurons) of the peri-infarct cortex, and the accumulation in parvalbumin-containing cells (presumably interneurons) was still evident (Figure 2 B). Additionally, MIF was found in GFAP^+ ^cells closely surrounding the infarct core, putative reactive astrocytes (Figure 2 B). In line with previous time-points, we did not observe MIF-ir in GST-π^+ ^oligodendrocytes at day 7 of recovery (Figure 2 C).

**Figure 3:**
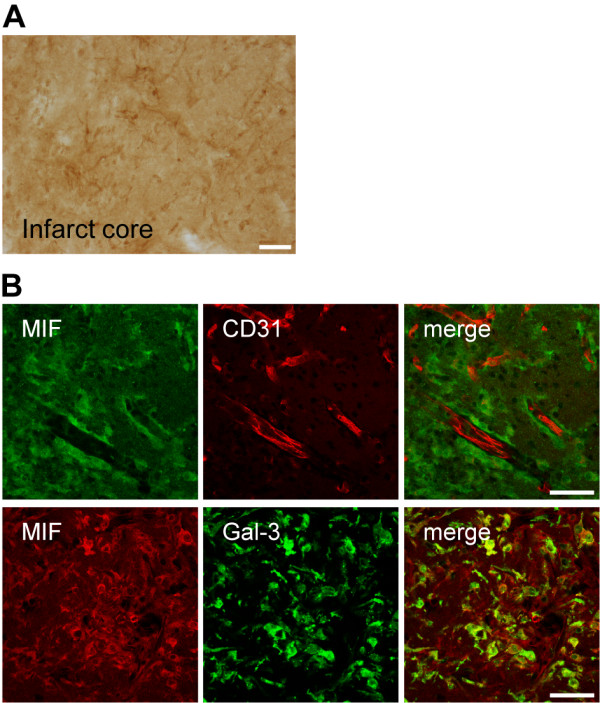
**Expression of MIF in the infarct core of C57BL/6 mice at day 7 after tMCAo**. **(A) **Representative bright field image of the striatal infarct core. Scale bar: 50 μm. **(B) **MIF is expressed in glia-like cells surrounding CD31^+ ^cells, upper panel. Scale bar: 50 μm. Co-localization of MIF (Cy3, red) with the microglial/macrophage protein galectin-3(Gal-3)/Mac-2 (Alexa488, green), lower panel. Scale bar: 50 μm.

Up to 72 h after stroke, the baseline cellular MIF-ir is lost within the infarct core, where merely a diffuse immunoreactivity pattern could be observed [29]. Between 72 h and 7 days after tMCAo, major changes in the expression of MIF occurred also in the infarct core (Figure 3 A). At day 7, and while still reduced in comparison to that in the peri-infarct region, MIF expression in the infarct core appeared in glial-like cells that surround CD31^+ ^endothelial cells, and in galectin-3^+ ^(Mac-2^+^) microglia/macrophages (Figure 3 B).

Antibody specificity for MIF was confirmed by the lack of immunoreactivity in the brains of MIF-KO mice, as shown before [29].

### Expression of inflammatory mediators in the brain and serum of WT and MIF-KO mice 48 h and 7 days after tMCAo

**Figure 4:**
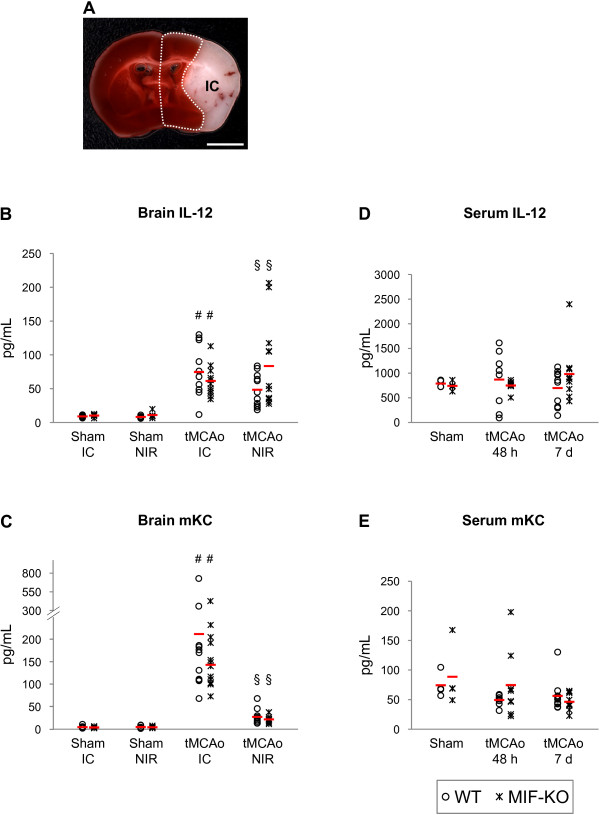
**Genetic deletion of MIF does not affect the cerebral up-regulation of IL-12 and mKC 48 h after tMCAo, in mice**. **A) **Schematic representation of the regions dissected from brains collected 48 h after surgery (sham and tMCAo): infarct core (IC, white) and non-infarct region (within dotted line).  Scale bar: 3mm. **B and D) **IL-12 and mKC protein levels in the cerebral IC and non-infarct region (NIR) of WT and MIF-KO subjected to sham-operation or tMCAo. **D and E) **IL-12 and mKC protein levels in the serum of WT and MIF-KO 48 h and 7 days after sham-operation or tMCAo. In B, C, D and E we present individual data points and the mean (dash). # p < 0.05 tMCAo IC versus Sham IC (for each genotype). § p < 0.05 tMCAo NIR versus Sham NIR (for each genotype).

We determined the expression of eight inflammatory mediators at the protein level in the brain and serum of WT and MIF-KO mice subjected to 45 min tMCAo and allowed to recover for either 48 h or 7 days. Mice sacrificed 48 h after sham-operation were included. Specifically, brains were collected 48 h and sera (and spleens) collected at both 48 h and 7 days after surgery. Table 2 summarizes the number of mice tested per experimental condition. Importantly, rCBF at MCA occlusion and reperfusion, body temperature, body weight and mortality were similar between genotypes. We dissected the cerebral infarct core (IC) and non-infarct region (NIR) as represented in Figure 4 A.

**Table 2:** Number of WT and MIF-KO mice used per experimental condition

	48 h		7 days
	
	Sham	tMCAo	tMCAo
WT	4-6	8-11	10

MIF-KO	4-6	8-12	9

Of the eight inflammatory mediators quantified (in brain and serum samples), only cerebral IL-12 and KC (also known as CXCL-1/CINC-1) protein concentrations differed significantly among the groups (Figure 4 B and 4C, respectively). We obtained a higher IL-12 protein concentration in the infarct core of WT mice subjected to tMCAo (74.6 ± 38.4 pg/mL, n = 11), than in the corresponding region of WT mice subjected to sham-operation (8.9 ± 1.8 pg/mL, n = 6), p = 0.001. A stroke-induced increase in IL-12 protein concentration occurred also in the non-infarct region of WT mice: 8.2 ± 1.8 pg/mL (sham, n = 6) and 48.4 ± 24.1 pg/mL (tMCAo, n = 11), p = 0.001. Similarly, IL-12 was up-regulated in the brain of MIF-KO mice following tMCAo versus sham-surgery: infarct core, 10.2 ± 2.2 pg/mL (sham, n = 6) and 61.5 ± 22.0 pg/mL (tMCAo, n = 12), p<0.0001; non-infarct region, 11.0 ± 4.5 pg/mL (sham, n = 6) and 83.5 ± 64.4 pg/mL (tMCAo, n = 12), p<0.05. However, we did not obtain significant differences between the two genotypes.

We found a robust up-regulation of KC in the infarct core of WT mice subsequent to tMCAo (212.1 ± 187.2 pg/mL), in comparison to sham-surgery (4.7 ± 3.3 pg/mL), p < 0.05. To a lower extent, KC also was induced in the non-infarct region of WT mice following stroke: 4.9 ± 3.3 pg/mL (sham) and 27.6 ± 16.6 pg/mL (tMCAo), p < 0.01. We detected similar values in brain samples from MIF-KO mice: infarct core, 4.0 ± 1.7 pg/mL (sham) and 167.1 ± 94.2 pg/mL (tMCAo), p < 0.001; non-infarct region, 4.5 ± 2.2 pg/mL (sham) and 22.0 ± 8.5 pg/mL (tMCAo), p < 0.0001. Hence, we did not find differences between genotypes.

The cerebral up-regulations of IL-12 and KC were not accompanied by significant changes in the respective serum concentrations 48 h and 7 days after stroke, and similar values were obtained in WT and MIF-KO mice per condition (Figure 4 D and 4E, accordingly).

**Figure 5:**
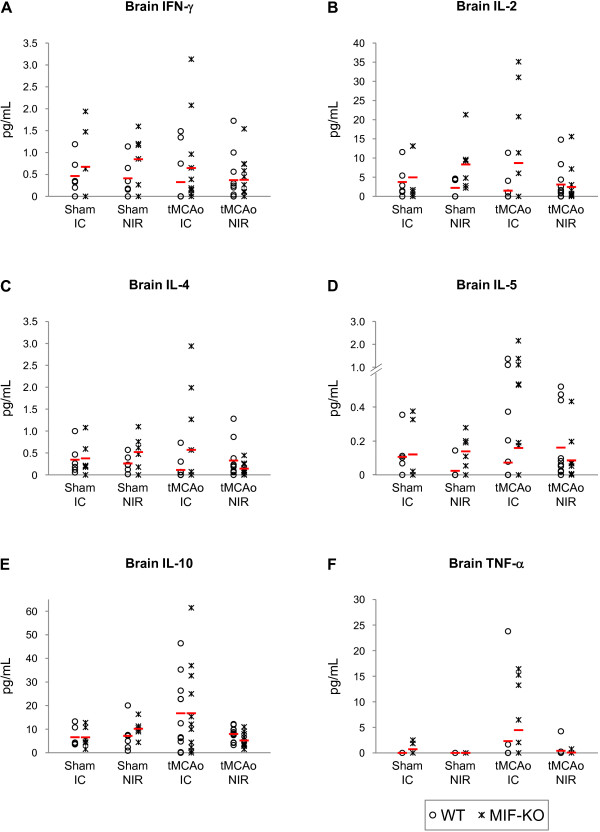
**IFN-β, IL-2, IL-4, IL-5, IL-10 and TNF-α protein levels in the brains of WT and MIF-KO mice 48 h after tMCAo**. Protein concentration in pg/mL; we present individual data points (as indicated in the figure) and the mean (dash). IC, infarct core; NIR, non-infarct region.

**Figure 6:**
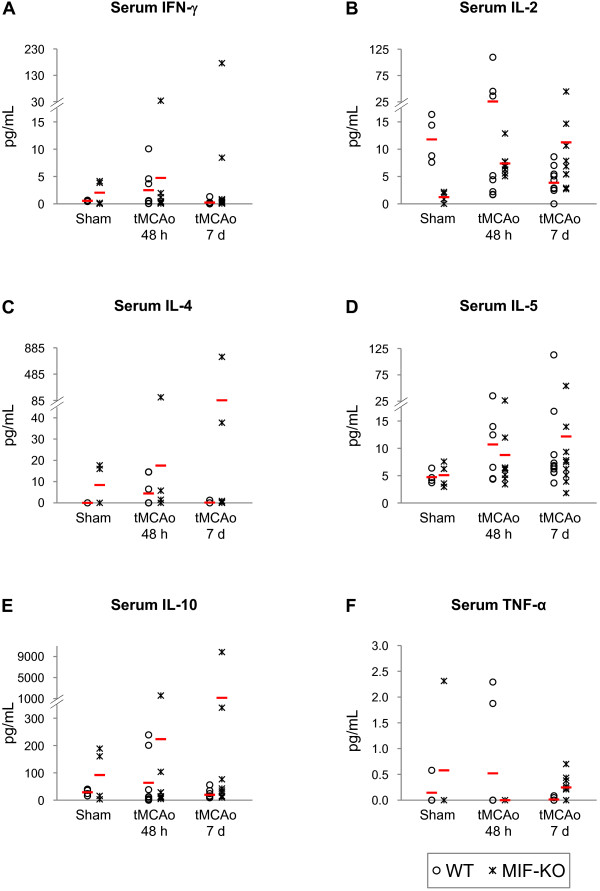
**Serum IFN-β, IL-2, IL-4, IL-5, IL-10 and TNF-α protein levels 48 h and 7 days after tMCAo, in WT and MIF-KO mice**. Protein concentration in pg/mL; we show individual data points (as indicated in the figure) and the mean (dash). IC, infarct core; P, peri-infarct region.

With respect to the protein levels of IFN-γ, IL-2, IL-4, IL-5, IL-10 and TNF-α in the brain, we did not find significant differences induced by stroke or by the deletion of MIF (Figure 5 A, B, C, D, E and 5F, respectively). Nevertheless, we obtained a strong trend towards a stroke-induced increase in the levels of IL-10 in the infarct core (p = 0.06). A strong trend towards an effect of stroke depending on the genotype on the levels of IL-10 in the non-infarct region also was obtained (p = 0.05). No differences in cytokines/chemokines levels were found in the serum (Figure 6 A, B, C, D, E and 6F).

### Spleen weight of WT and MIF-KO mice 48 h and 7 days after tMCAo

**Figure 7:**
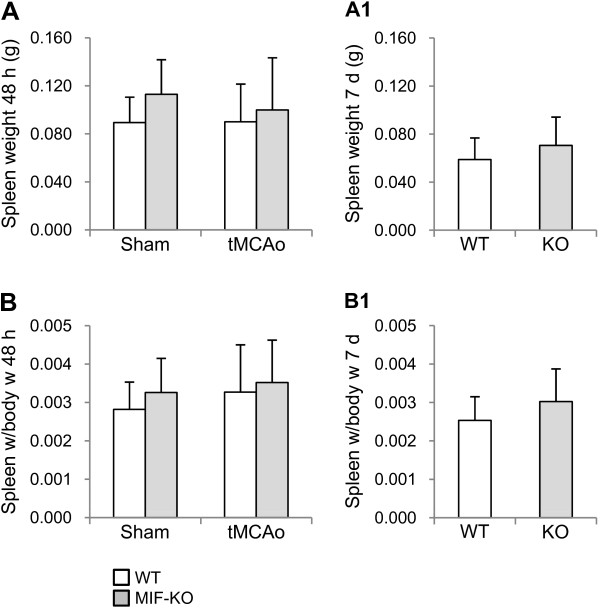
**Spleen weight of WT and MIF-KO mice up to 7 days after tMCAo**. Spleen weight (in g; mean ± STD) of WT and MIF-KO mice 48 h after either sham-surgery or tMCAo (A) and 7 days after tMCAo (A 1), and respective spleen weight (g)/body weight (g) ratios (B and B 1).

We further studied a putative effect of MIF on the spleen weight of mice during the first week post-stroke. The spleen weights of sham-operated WT (n = 6) and MIF-KO (n = 6) mice were not significantly different (Figure 7 A): 0.089 ± 0.021 g and 0.133 ± 0.029 g, respectively. At 48 h after tMCAo, the spleen weight did not differ significantly between WT (0.090 ± 0.031 g, n = 11) and MIF-KO (0.100 ± 0.043 g, n = 12) mice (Figure 7 A). At this time point post-occlusion, the spleen weight was similar to the baseline (i.e., sham-operated animals), for both genotypes. Moreover, the deletion of *Mif *in mice did not affect spleen weight up to 7 days after tMCAo (Figure 7 A1): 0.059 ± 0.013 g, WT mice (n = 13); 0.071 ± 0.024 g, MIF-KO mice (n = 9).

As previously indicated we did not detect significant differences in body weight before and body weight/weight loss after surgery between genotypes. Yet, we normalized spleen weight to body weight. Also in this case, we did not obtain a significant difference between genotypes (Figure 7 B and 7B1).

### GFAP immunoreactivity in the cerebral peri-infarct region of WT and MIF-KO mice 7 days after tMCAo

**Figure 8:**
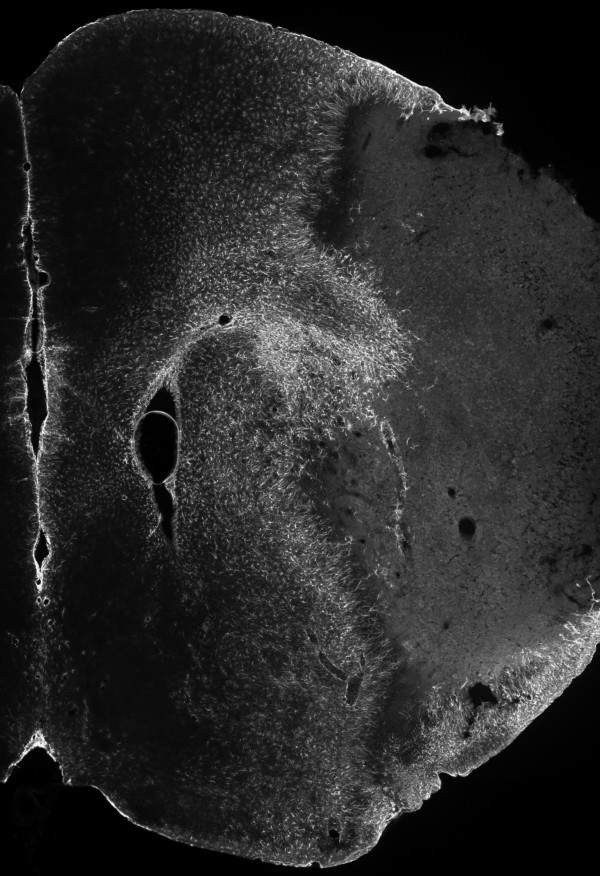
**Cerebral GFAP immunoreactivity at day 7 post-tMCAo, in mice**. Composite image representing the obtained GFAP immunoreactivity (Cy3, gray-scale) within the right brain hemisphere 7 days after a 45 min occlusion of the right middle cerebral artery, in C57BL/6 mice (epifluorescence micrographs).

The up-regulation of intermediate filament proteins, such as GFAP, is a hallmark of reactive astrogliosis, including glial scar formation bordering the infarct core after stroke [10]. To determine if MIF affects the extent of reactive astrogliosis, we studied GFAP immunoreactivity (GFAP-ir) in the brains of WT and MIF-KO mice, 7 days post-occlusion. Figure 8 exemplifies the obtained cerebral GFAP-ir within the ispilateral hemisphere. We did not detect a critical difference between the WT (n = 12-13) and MIF-KO (n = 9) groups with respect to all of the quantified parameters, i.e., area of GFAP-ir (excluding the hippocampus), signal intensity within the GFAP^+ ^area, signal intensity within the ipsilateral hippocampus, and width of GFAP-ir. We did not detect overall or brain level-specific differences between genotypes (data not shown).

### Number of CD74^+ ^cells in the cerebral infarct core 7 days after tMCAo in WT and MIF-KO mice

Previous studies suggest that MIF modulates the infiltration of inflammatory/immune cells into the brain parenchyma under pathological conditions. It is known that MIF acts on the cell surface receptor CD74 [34], expressed in antigen-presenting cells, or APC's [35]. We found that CD74^+ ^cells, putative APCs, accumulate in the brains of C57BL/6 mice during the first week after stroke [36]. We thus hypothesized an effect of the genetic deletion of MIF in mice on the number of CD74^+ ^cells within the brain 7 days post-tMCAo.

**Figure 9:**
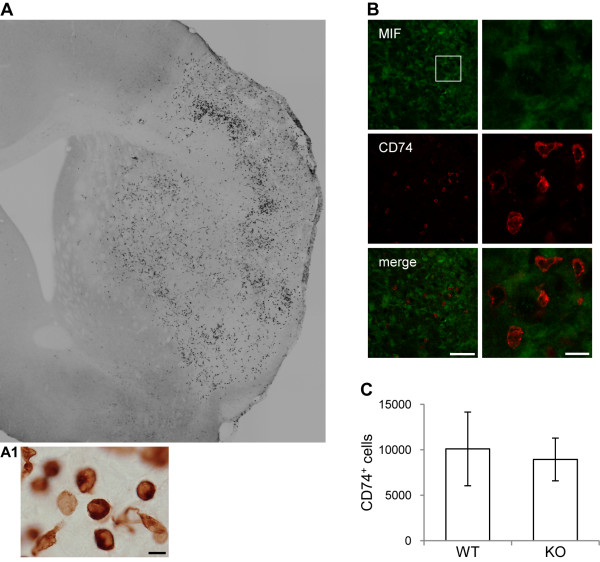
**Number of CD74**^**+ **^**cells in WT and MIF-KO brains 7 days after tMCAo**. **(A and A1) **Epifluorescence micrographs. **(A) **Representative composite image of CD74 immunoreactivity in the mouse brain 7 days post-occlusion (WT). Note the presence of CD74^+ ^cells essentially within the infarct core. **(A1) **Higher magnification micrographs denote the morphology of CD74^+ ^cells. Scale bar: 10 μm. **(B) **Confocal images. Lack of co-localization MIF (Alexa 488, green)/CD74 (Cy3, red) in the infarct core. Scale bars: 50 μm and 10 μm (lower and higher magnification, respectively). **(C) **Number of CD74^+ ^cells detected per ipsilateral brain hemisphere (sum of the analyzed brain levels) - WT and MIF-KO mice 7 days post-occlusion of the MCA (mean ± STD).

We first examined in more detail the expression of CD74 in the mouse brain (WT) 7 days after tMCAo (using immunohistochemistry). CD74^+ ^cells appeared essentially in the injured brain hemisphere, clearly within the infarct core, determined by the lack of NeuN immunoreactivity (Figure 9). While varying among individuals (n = 15), the distribution of CD74^+ ^cells often comprised a clear higher density rim within the cortex (as exemplified in Figure 9 A); in some cases, a complete high-density rim shaping the infarct core, but within its boundaries could be found. Moreover, CD74^+ ^cells exhibited diverse morphologies (Figure 9 A1). Up to 7 days after tMCAo, we could not find CD74 expression in brain cells. We found CD74^+ ^cells in the cerebral infarct core as early as 3 days, but not to the same extent as at 7 days post-tMCAo (data not shown). Although CD74^+ ^cells were at 7 days in close vicinity with MIF^+ ^cells, we did not find CD74/MIF double-labeled cells (Figure 9 B).

Finally, we found a similar number of CD74^+ ^particles per brain (that we refer to as CD74^+ ^cells, for simplification) in WT (n = 12) and MIF-KO mice (n = 9): 10111 ± 4058 and 8947 ± 2346, respectively (p = 0.45), (Figure 9 C). Additionally, the area of CD74-ir (or encircled area) per section was similar between the groups. Importantly, we found no difference in the *particles area *per section, neither in the fraction *particles area/encircled area *per section between groups. The CD74-ir (signal intensity) did not differ between genotypes.

## Discussion

We will discuss the primary findings of the study: the expression of MIF in the mouse brain is highly dynamic (1); the deletion of *Mif *in mice does not affect the expression of Th1/Th2 cytokines in the brain and serum (2), spleen weight (3), reactive astrogliosis (4) and number of CD74^+ ^cells in the brain (5); after stroke.

### MIF and the expression of Th1/Th2 cytokines in the mouse brain and serum after stroke

We previously found that disruption of *Mif *in mice (on a C57BL/6 background) results in a smaller cerebral infarct volume and in a lower extent of sensory-motor deficits during the first week after stroke. In particular, MIF-KO mice performed significantly better than WT littermates at both the rotating pole and grip strength tests 48 h after tMCAo. At this time point, i.e., 48 h after tMCAo, the expression of IL-1β increased in the cerebral infarct core (but not in the serum). Importantly, we obtained similar IL-1β protein levels in the brain and serum of WT and MIF-KO mice [29]. Here, we analyzed the expression of eight additional Th1/Th2 cytokines, also at the protein level. While stroke resulted in a significant increase in IL-12 and mKC protein levels in the brain [37] (infarct core and non-infarct region), we did not detect an effect of the genotype on the expression of the inflammatory mediators analyzed, in the brain and serum.

It has become a general concept that MIF promotes the production/release of pro-inflammatory cytokines upon inflammatory stimulus [19]. For instance, MIF-KO mice (C57BL/6 x Sv129, males) have lower circulating IFN-γ, IL-12 and TNF-α protein levels (diminished Th1 response) upon *Salmonella typhimurium *infection [38]. Of particular relevance, the expression of several cytokines in the brain, including IFN-γ, IL-12 and TNF-α, at mRNA level is lower is MIF-KO mice (BALB/C females) upon West Nile Virus infection [26]. This was accompanied by a decrease in TNF-α (protein) in the serum and by a decrease in splenic IL-12 (mRNA). The authors discuss that a decrease in cytokine expression in the brain may however result from a decreased viral neuroinvasion. Recently and apart from infection, Gao *et al*. showed a decrease in classical pro-inflammatory cytokines, including IL-1β and TNF-α, as well as an increase in IL-10 protein levels in the myocardium of MIF-KO versus WT mice (on a C57BL/6 background, male) following 60 min ischemia and 24 h reperfusion [39]. Here, it is also possible that the altered cytokine production depends primarily on a smaller infarct obtained for MIF-KO mice.

In fact, the exact pattern of modifications in cytokine production in the presence or absence of MIF varies across studies. For instance, IL-1β can be down-regulated [39], up-regulated [38] or unchanged [26] in MIF-KO mice upon inflammatory stimulus. Moreover, in a mouse model of multiple sclerosis, experimental autoimmune encephalomyelitis, no significant differences in the peripheral levels of IL-10, IL-17, or IFN-γ between MIF-KO mice (on a C57BL/6 background, males) and WT controls were observed [27]. In line with our results, the authors suggest that MIF is not required for Th1-mediated immunity, and that the deletion of MIF does not result in a shift to a Th2 response.

Despite differences in experimental models, it is becoming increasingly evident that the effect of MIF depends on the type of cellular stimulus, spatial-temporal context of MIF expression, and respective concentration range [40], and eventually on the disease stage [38]. Our data suggests that while promoting neuronal death and aggravating neurological deficits, MIF expression is not essential in driving the production of Th1/Th2 cytokines post-stroke, at least at the time-points studied.

### Spleen weight after tMCAo in mice

We did not detect a difference in spleen weight between WT and MIF-KO mice under control conditions (sham-operations) or after tMCAo, and we are not aware of previously reported differences in spleen weight or histological parameters caused by the deletion of MIF [38]. The apparent reduction in spleen weight at day 7 post-stroke versus sham is in convergence with previous studies [18]. However, stroke-induced splenic atrophy was no longer apparent after the normalization spleen weight/body weight, indicating that weight loss correlates with and may even precede spleen atrophy after experimental stroke.

### MIF and reactive gliosis

We observed that MIF is expressed in GFAP^+ ^cells surrounding the infarct core (or reactive astrocytes) 7 days after tMCAo in C57BL/6 mice. This is line with our previous observation that MIF is highly expressed in reactive astrocytes (of the peri-infarct region) at 5 days after permanent MCAo in rats [30]. These results indicate that the up-regulation of MIF is part of a conjunct of molecular alterations that is on the basis of reactive astrogliosis and eventually glial scar formation after stroke [10]. Moreover, factors known to induce MIF expression, such as hypoxia-related factors [28,41] and cytokines [42], also are known to induce reactive astrogliosis, and these two processes may be highly inter-connected.

We reported previously that housing rats in an enriched environment 2 to 5 days subsequent to permanent MCAo, a paradigm that dramatically improves sensory-motor function without altering infarct volume, results in a down-regulation of MIF protein levels around the infarct core (at 5 days post-stroke) [30]. Importantly, the down-regulation of MIF was not secondary to a decrease in reactive astrogliosis/glial scar formation, given by an unchanged GFAP protein level between housing conditions. These results implicate that a lower astrocytic MIF content correlates with an improved sensory-motor function in rats, 5 days after stroke. Here, we found a similar GFAP-ir in the brains (damaged hemisphere) of WT and MIF-KO mice at 7 days post-tMCAo further suggesting that MIF is not essential for but is part of reactive astrogliosis. Overall, a reduced expression or abrogation of MIF in astrocytes may underlie an improved functional recovery, particularly long-term, after stroke. Although previously understated, molecular alterations in reactive astrocytes are believed to affect among others neuronal function, microglial function and immune cell invasion, and thereby the outcome following stroke [43].

We previously observed that MIF-KO mice show a similar CD68 immunoreactivity, but a higher Gal-3 immunoreactivity within the injured brain hemisphere 7 days following experimental stroke, in comparison to WT littermates [29]. These results suggest that MIF modulates the microglia/macrophages response following stroke. This may occur via an increase in the expression of Gal-3, an increase in the proliferation of Gal-3^+ ^cells and an increase in the infiltration of monocytes into the brain parenchyma [26,27]. Macrophages are a known source of MIF [42]. Here, we found that MIF is expressed in Gal-3^+ ^cells, microglia/macrophages, within the infarct core 7 days after tMCAo.

The particular expression of MIF in glial cells may underlie the modifications in the microglia/macrophages response here in cause. However, the exact mechanism(s) of action of MIF in astrocytes and in microglia, or at the interface among neurons, astrocytes, microglia and immune cells remain(s) to be identified.

### MIF and immune cell recruitment

MIF may promote [26,27] or inhibit [25] immune cell infiltration into the brain parenchyma under pathological conditions. In particular, Bacher *et al*. (2002) proposed that the induction of MIF in end-feet astrocytes at the blood-brain barrier (BBB) inhibits macrophage infiltration into the brain upon Borna Disease Virus infection [25]. Besides the accumulation in astrocytes and microglia, we observed that MIF appears in glial-like cells surrounding blood vessels within the infarct core 7 days after tMCAo. The particular accumulation of MIF in glial-like cells surrounding blood vessels may influence immune cell infiltration into the brain parenchyma [44]. Here, we further evaluated the effect of genetic deletion of MIF in mice on the number of CD74^+ ^cells within brain. First, CD74, also known as major histocompatibility complex (MHC) class II-associated invariant chain (li), is a cell surface MIF-receptor [34]. Second, CD74 may play a role in CNS injury [45]. Third, we previously found a lower number of CD74^+ ^cells in the brains of legumain-deficient mice, when compared to the brains of WT littermates, suggesting that either the expression of this protein or more likely the recruitment of these cells is regulated by the expression of inflammatory/immune cues after stroke [36]. CD74 is not expressed in brain cells, and the absence of MIF did not affect the number of CD74^+ ^cells within the injured brain hemisphere, suggesting that the MIF-CD74 signaling is not involved in brain function after stroke.

The overall lack of an effect of the genetic deletion of MIF in mice suggests that alternative inflammation-related and/or unrelated mechanisms may be involved. Based on previous *in vivo *and *in vitro *experiments [29,31,32], it is tempting to speculate that the upregulation of MIF in neurons underlies the observed deleterious effects, at least during the first week post-stroke in mice. Beyond an eventual neuroimmunomodulatory role, it is possible that MIF acts primarily as neuromodulator in the brain.

## Conclusion

Our data indicates that MIF does not affect main components of the inflammatory/immune response following stroke. The expression and regulation of MIF in neurons may be primordial in the development of the infarct and neurological deficits, whereas the (over)expression in astrocytes and microglia, and the modulation of microglia/macrophages response may further depress the recovery of lost neurological function. The broad spectrum of MIF actions and the complexity of MIF expression in the brain post-stroke challenge the identification of the exact mode or modes of action of MIF in cerebral ischemia.

## List of abbreviations used

(CD): cluster of differentiation; (Gal-3): galectin-3; (GFAP): glial fibrillary acidic protein; (IC): infarct core; (IL): interleukin; (ir): immunoreactivity; (MIF): macrophage migration inhibitory factor; (MIF-knockout, MIF-KO): *Mif*^-/-^; (wild-type, WT): *Mif*^+/+^; (mKC): mouse keratinocyte-derived cytokine; (PV): parvalbumin; (NIR): non-infarct region; (^+^): -positive; (tMCAo): transient middle cerebral artery occlusion.

## Competing interests

The authors declare that they have no competing interests.

## Authors' contributions

ARI and TD conceived the study. ARI designed and performed the experiments, analyzed the data, and wrote the manuscript. TD designed experiments, performed data analysis and edited the manuscript. RB provided MIF-KO mice and edited the manuscript. All the authors approved the present manuscript.
